# Pardaxin Activates Excessive Mitophagy and Mitochondria-Mediated Apoptosis in Human Ovarian Cancer by Inducing Reactive Oxygen Species

**DOI:** 10.3390/antiox10121883

**Published:** 2021-11-25

**Authors:** Yen-Po Chen, Po-Chang Shih, Chien-Wei Feng, Chang-Cheng Wu, Kuan-Hao Tsui, You-Hsien Lin, Hsiao-Mei Kuo, Zhi-Hong Wen

**Affiliations:** 1 Department of Marine Biotechnology and Resources, National Sun Yat-Sen University, Kaohsiung 80424, Taiwan; D075020002@nsysu.edu.tw (Y.-P.C.); po-chang.shih.14@ucl.ac.uk (P.-C.S.); nhk@ngh.com.tw (C.-C.W.); 2 Department of Obstetrics and Gynecology, Kaohsiung Armed Forces General Hospital, Kaohsiung 80284, Taiwan; 3 Department of Neurosurgery, Kaohsiung Chang Gung Memorial Hospital, Kaohsiung 83301, Taiwan; 4 Department of Obstetrics and Gynecology, Kaohsiung Medical University Hospital, Kaohsiung Medical University, Kaohsiung 80756, Taiwan; 1080532@kmuh.org.tw; 5 Department of Obstetrics and Gynecology, Zouying Branch of Kaohsiung Armed Forces General Hospital, Kaohsiung 81342, Taiwan; 6 Department of Obstetrics and Gynecology, Kaohsiung Veterans General Hospital, Kaohsiung 81341, Taiwan; khtsui@vghks.gov.tw; 7 Department of Obstetrics and Gynecology, Institute of Clinical Medicine, National Yang-Ming University, Taipei 11221, Taiwan; 8 Department of Internal Medicine, Kaohsiung Municipal Ta-Tung Hospital, Kaohsiung 80145, Taiwan; hugoyl@kmu.edu.tw; 9 Department of Medicine, College of Medicine, Kaohsiung Medical University, Kaohsiung 80708, Taiwan; 10 Center for Neuroscience, National Sun Yat-Sen University, Kaohsiung 80424, Taiwan; 11 Institute of Medical Science and Technology, National Sun Yat-Sen University, Kaohsiung 80424, Taiwan

**Keywords:** pardaxin, mitophagy, mitochondria, reactive oxygen species, apoptosis, natural product, ovarian cancer, oxidative phosphorylation, autophagosome, mitochondrial membrane potential

## Abstract

Most ovarian cancer (OC) patients are diagnosed with stage III or higher disease, resulting in a poor prognosis. Currently, paclitaxel combined with carboplatin shows the best treatment outcome for OC. However, no effective drug is available for patients that do not respond to treatment; thus, new drugs for OC are needed. We evaluated the antimicrobial peptide, pardaxin, in PA-1 and SKOV3 cells. Pardaxin induced apoptosis as determined by MTT and TUNEL assays, as well as activation of caspases-9/3, Bid, t-Bid, and Bax, whereas Bcl-2 was downregulated. The IC_50_ values for pardaxin were 4.6–3.0 μM at 24 and 48 h. Mitochondrial and intracellular reactive oxygen species (ROS) were overproduced and associated with disrupted mitochondrial membrane potential and respiratory capacity. Additionally, the mitochondrial network was fragmented with downregulated fusogenic proteins, MFN1/2 and L-/S-OPA1, and upregulated fission-related proteins, DRP1 and FIS1. Autophagy was also activated as evidenced by increased expression of autophagosome formation-related proteins, Beclin, p62, and LC3. Enhanced mitochondrial fragmentation and autophagy indicate that mitophagy was activated. ROS-induced cytotoxicity was reversed by the addition of N-acetylcysteine, confirming ROS overproduction as a contributor. Taken together, pardaxin demonstrated promising anticancer activity in OC cells, which warrants further preclinical development of this compound.

## 1. Introduction

Ovarian cancer (OC) is the fifth leading cause of cancer-related deaths in females [1] with over 22,000 new cases each year and 14,000 deaths occurring [2], with the epithelial type OC being the most common [3]. Epithelial OC may be further categorized into primary serous, endometrioid, mucinous, and clear cell histologic subtypes [4]. Conventional standard treatment for OC includes a combination of cytoreductive surgery and chemotherapy: tumor debulking surgery, surgical staging, and subsequent platinum-based chemotherapy [5]. Despite a high response rate, tumors eventually develop resistance and are refractory to further treatment. Therefore, the development of new treatments for OC patients is a priority.

Reactive oxygen species (ROS) are of particular interest since their deleterious effects in cell physiology and pathology were reported by Droge et al. [6]. ROS include superoxide anion radicals (O_2_^•−^), hydroxyl radicals (^•^OH), hydrogen peroxide (H_2_O_2_), and singlet oxygen [7]. In cells, mitochondria are the primary site of ROS generation because of their respiratory and energy production cascade. At low concentrations, ROS mediate various biological functions including cellular redox signaling, whereas at high concentrations, they are associated with cytotoxic mechanisms [8]. To prevent cellular damage and apoptosis caused by ROS, cells contain antioxidant enzymes, such as superoxide dismutases, to detoxify ROS [9,10]. Over the years, the generation of ROS has been studied as a treatment for cancer [11].

Mitochondria are double-membraned organelles that vary considerably in size and structure [12]. They are highly dynamic organelles that have the ability to change size, shape, and position in seconds [13]. Many of these changes are associated with the ability of mitochondria to undergo a highly coordinated process of fission or fusion [14], which regulates their overall morphology. The dynamic processes of fission and fusion are essential, which require various proteins to coordinate, for example, mechanical proteins that alter mitochondrial membrane structurally and adapter proteins that mediate the interactions of these mechanical proteins with intracellular organelles. MFN1, MFN2, and OPA1 are fusion proteins, while FIS1 and DRP1 are fission proteins, all of which play a critical role in the dynamic changes of mitochondria [4]. Studies have reported that high levels of ROS produced in cells following drug exposure can yield cytotoxic effects and induce apoptosis in malignant cells by disrupting the mitochondrial membrane and oxidative phosphorylation [8,15,16]. Mitochondria are also involved in the induction of intrinsic apoptosis with caspases 9 and 3 being activated. The engagement of the mitochondria-involved apoptotic pathway and caspase 9 activation are typically considered a point of no return in cell death signaling [17].

Autophagy is a cytoprotective mechanism that involves homeostatic functions such as cytoplasm, protein, and organelle turnover [18]. Fundamental autophagy is a highly regulated process in which trimmed imbalance is potentially detrimental to cell survival. Autophagy involves the initial formation of an isolation membrane (also known as a phagocytic bubble), which extends and closes around the cytoplasmic cargo to form a double-membraned autophagosome, followed by fusion with lysosomes to form autologous lysosomes where substance degradation occurs [19]. Many autophagy-related proteins are involved in this dynamic and highly regulated process. In particular, the mammalian homologs, Beclin 1 and LC3, are necessary for the formation of the isolation membrane of autophagosome [20]. Under normal conditions, autophagy serves as a homeostatic mechanism that initiates degradation of damaged proteins and organelles. Interestingly, autophagy in cancer is bipolar that exhibits both tumor-survival and tumor-suppressive roles, and the type of roles depends on tumor type, stage, and genetic background. Mitophagy, also known as macroautophagy, is a specific form of autophagy that degrades damaged mitochondria. Interestingly, excessive mitophagy can activate apoptotic cell death, which is one area of interest for anticancer drug development [21].

Oceans are a treasure trove for developing novel chemotherapeutics, since they account for nearly 70% of the total global area and contain a diversity of ecosystems much greater than that of terrestrial systems. Antimicrobial peptides (AMPs) represent a large family of marine compounds that exhibit an overall high efficacy against Gram-positive and Gram-negative bacteria [22,23,24,25]. MSPs are a group of AMPs containing approximately 25–80 amino acids [26,27]. Recently they have demonstrated activity against cancer, such as breast cancer [28], human cervical cancer [29,30], fibrosarcoma [31], and other types [31,32]. Pardaxin was originally isolated from the sole species, *Pardachirus marmoratus*. It contains a helix–hinge–helix structure that is common in peptides able to lyse mammalian and bacterial cells [33], with its N-terminal α-helix for insertion into the lipid bilayer of a cell [34]. In this study, we evaluated pardaxin to two different OC cells, PA-1 and SKOV3, to determine its anticancer activity. We also examined its underlying mechanism of action at the cellular and molecular level.

## 2. Materials and Methods

### 2.1. Reagents

PBS (phosphate buffered saline) was used to prepare pardaxin stock solution which was stored at −20 °C. A powder of 3-(4,5)-dimethylthiazol(-z-y1)-3,5-diphenyltetrazolium bromide (known as MTT) and dimethyl sulfoxide (DMSO) reagent were acquired from Sigma-Aldrich (St. Louis, MO, USA). *N*-acetylcysteine (NAC) and MTT, respectively, were prepared in DMSO and PBS as stock solutions which were then freshly diluted in the proper media at concentrations of interest. An In Situ Cell Death Detection Kit, Fluorescein, used in the Terminal Transferase-mediated dUTP Nick-End Labeling (TUNEL) method, was acquired from Roche Life Science (Penzberg, Upper Bavaria, Germany). DAPI (40,6-Diamidino-2-Phenylindole, Dilactate), FITC-Annexin V/PI assay kit, the chloromethyl derivative of 2,7-dichlorodihydro- fluorescein diacetate (CM-H_2_DCFDA), MitoSOX^TM^ Red, and JC-1 were acquired from Molecular Probes, Inc. (Eugene, OR, USA). A Seahorse XF Cell Mito Stress Test kit that contained enzymatic inhibitors carbonyl cyanide-4-(trifloromethoxy)phenylhydrazone (FCCP), oligomycin, and rotenone, were acquired from Agilent Technologies (Santa Clara, CA, USA). The plasmid, pcDNA 3.1(+)-DsRed2-Mito-7, for the study of mitochondrial morphology was acquired from Addgene Corporation (Watertown, MA, USA). CellROX^®^ was acquired from Life Technologies (Carlsbad, CA, USA). NAC was acquired from Santa Cruz Biotechnology (Dallas, Texas, USA), while DRAQ7^TM^ was acquired from Abcam (Cambridge, UK).

### 2.2. Cell Lines and Maintanance

The human OC cell line, PA-1 cells (ATCC^®^ CRL-1572™, *Homo sapiens* ovary epithelial teratocarcinoma), were cultured with the Eagle’s Minimum Essential medium. The human OC cell line, SKOV3 cells (ATCC^®^ HTB-77™, *Homo sapiens* ovary epithelial adenocarcinoma) were cultured with the RPMI 1640 medium (Life Technologies^TM^). Both media were supplemented with 10% (*v/v*) heat-inactivated fetal bovine serum (Gibco^®^), 2 mM glutamine, 50 U/mL penicillin and 50 mg/mL streptomycin (Sigma-Aldrich^®^). The cell lines were cultured under a humidified atmosphere of 5% CO_2_ mixed in 95% air at 37 °C, and they were examined under a Leica DMI 3000B phase contrast microscope (Leica Microsystems, Wetzlar, Germany) regularly. After microscopic examination, these cells were used for the following experiments.

### 2.3. MTT-Based Cell Viability Determination

A MTT-based assay was performed to assess cell viability as described in the literature [35]. A density of 3 × 10^4^ cells/well were seeded in transparent 96-well plates (Nunc, Roskilde, Denmark) and placed overnight in a 37 °C incubator equipped with a humidified atmosphere mixing with 5% CO_2_ and 95% air. The OC cells were challenged with 0, 0.01, 0.1, 1, 2.5, 5, 10, and 20 μΜ of pardaxin for 24 or 48 h. Subsequently, the drug-containing medium was replaced with the MTT solution, and the OC cells were further incubated in the incubator for 4 h to allow MTT being reduced to an insoluble formazan crystal through viable cell dehydrogenases. Each concentration was performed in triplicate. The Leica DMI 3000B phase contrast microscope was used to observe the cells during the experimentation. DMSO was used to redissolve the formazan crystal. Absorbance at 570 nm was recorded using a Dynatech Laboratories ELISA reader (Chantilly, VA, USA) for calculating cell viability, and the results are showed as mean ± SD. 

### 2.4. Terminal Deoxynucleotidyl Transferase-Mediated dUTP (TUNEL) Assay

The TUNEL assay was conducted to probe DNA fragmentation using fluorescence microscopy. The terminal deoxynucleotidyl transferase (TdT) included in the assay kit can catalyze fluorescein-dUTP incorporation at free 3′-hydroxyl ends of the fragmented DNA, allowing it to be detected. In brief, the OC cells plated on sample slides with pardaxin at 0, 0.1, 1, 2.5, and 5 μΜ for 24 h were fixed with pH 7.4, 4% methanol-free formaldehyde for 5 min on ice. Following ice-cold PBS rinse, the *In Situ* Cell Death Detection Kit, Fluorescein was used for TUNEL analysis as per manufacturer’s user manual. Afterwards, green fluorescent TUNEL-positive cells were detected by an immunofluorescence microscope acquired from Lecia Microsystems (Wetzlar, Germany). In addition, the TUNEL-positive cells showing green fluorescence were spotted with the assistance of DAPI staining and by cellular morphology. The sample slides were observed using a fluorescence-detectable microscope, and green fluorescence was fixed at 520 nm. A SPOT CCD RT-slider integrating camera (Diagnostic Instruments, Sterling Heights, MI, USA) was used to captured images. The cells stained green suggested apoptotic cells, while cells fluoresced blue represented DNA fragmentation.

### 2.5. Staining of FITC-Annexin V/Propidium Iodide (PI)

To detect mode of cell death, FITC-Annexin V/PI method was used in flow cytometric analyses, of which FITC is a fluorophore. Annexin V-positive signals are an indicator of cellular apoptosis, while PI-positive indicates necrotic or late apoptotic cells, due to disrupted integrity of cell and nuclear membranes. Viable cells are not responsive to both staining probes. Briefly, the OC cells were challenged with pardaxin at 0, 0.1, 1, 2.5, and 5 μΜ for 24 h, and they were harvested and cold PBS washed. After resuspending the cells in 1X binding buffer, a solution containing a concentration of 6 × 10^5^ cells/mL was prepared. One hundred (100) μL of the solution (6 × 10^4^ cells) were then transferred to a 5 mL tube. The resulting samples were subjected to FITC-Annexin V labeling as per manufacturer’s user manual. In brief, 100 μL of 1X Annexin V binding buffer from BD Biosciences (Franklin Lakes, NJ, USA) were used to resuspend the cells which were then fluorescently labeled by mixing 5 mL of FITC-Annexin V and 5 mL of 1 mg/mL PI (BD Biosciences) to each sample. After gentle mixing and incubation for 30 min at room temperature in a dark room, 1 mL of the 1X binding buffer was added to each sample. The yielded samples were analyzed using a flow cytometer (Beckman Coulter, Southfield, MI, USA) with Cell Lab Quanta™ SC analysis software. At least 10^4^ cells/sample were analyzed.

### 2.6. Detection of Cellular ROS

The following procedures were conducted as described in the literature [36,37]. Intracellular ROS were determined by hydrogen peroxide (H_2_O_2_) level using a chemical probe CM-H_2_DCFDA. In the presence of H_2_O_2_, cell-permeable CM-H_2_DCFDA is processed by intracellular esterases and oxidized into a fluorescent adduct of 2,7-dichlorodifluorescein (DCF) which is detectable using flow cytometry. Prior to use, the CM-H2DCFDA probe was protected from air. Briefly, 6 × 10^5^ cells/well were seeded in 6-well plates with the specified media and placed for 16–18 h for cell attachment on the plate bottom in the 37 °C incubator equipped with a humidified atmosphere mixing with 5% CO_2_ and 95% air. After washing with PBS, the culture media were replaced with pardaxin-containing media at concentrations of 0–5 μΜ and incubated for 4 h at 37 °C. Subsequently the cells were incubated with 5 μΜ CM-H_2_DCFDA for additional 30 min at 37 °C. Following washing and resuspending in PBS, the Beckman Coulter cytometer was used to detect fluorescence signals with excitation/emission at 495/520 nm and analyzed with Cell Lab QuantaTM SC software. At least 10^4^ cells/sample were analyzed.

### 2.7. Detection of Mitochondrial ROS

The following procedures were conducted as described in the literature [36,37]. Mitochondrial superoxide (O2^•−^) level was analyzed by cell-permeable MitoSOX^TM^ Red (Molecular Probes, Inc), a redox-sensitive dye believed to specifically target mitochondrial matrix, and red fluorescence is emitted upon oxidation. A density of 6 × 10^5^ cells/well were seeded with the specified media and placed for 16–18 h for cell attachment on the plate bottom in the 37 °C incubator equipped with a humidified atmosphere mixing with 5% CO_2_ and 95% air. After being treated with pardaxin at 0–5 μM for 4 h at 37 °C, the cells were rinsed with PBS once. The MitoSOX^TM^ Red at 10 mM was added to the OC cells which were incubated for 10 min at 37 °C, rinsed, and redissolved in PBS. The cytometer of Beckman Coulter and Cell Lab Quanta™ SC software was used for analysis. The absorption/emission wavelengths were set at 510/580 nm. At least 10^4^ cells/sample were analyzed.

### 2.8. CellROX^®^ Green Staining

CellROX^®^ Green is a fluorogenic dye that measures oxidative stress in live cells. The cell-permeable probe emits weak fluorescence under a reduced state, whereas upon oxidation by ROS, it exhibits bright green fluorescence and subsequently binds to DNA with absorption and emission peaked at ~485 and 520 nm, respectively. Before use, the reagent was placed at −20 °C and protected from light. In brief, a density of 6 × 10^5^ cells/plate were seeded with the specified media and placed for 16–18 h for cell attachment on the plate bottom in the 37 °C incubator equipped with a humidified atmosphere mixing with 5% CO_2_ and 95% air. After being treated with pardaxin at 0–5 μM for 4 h, the OC cells were rinsed with PBS once. CellROX^®^ Green at 5 mM was added to the cells which incubated for 10 min at 37 °C, rinsed, and redissolved in PBS. The cytometer of Beckman Coulter and Cell Lab Quanta™ SC software was used for analysis. At least 10^4^ cells/sample were analyzed.

### 2.9. JC-1 Staining for Measuring Mitochondrial Membran Potential

The following procedures were conducted as described in the literature [37]. A fluorescent dye, JC-1, was used for detecting mitochondrial membrane depolarization. The positively charged, monomeric form of JC-1 dye exhibits accumulation in negatively charged mitochondria at a potential-dependent manner, as indicated by green fluorescence emission at ~529 nm, which is shifted to red peaked at ~590 nm with a concentration-dependent generation of red fluorescent J-aggregates upon changes in mitochondria health. On this basis, a decrease in the red/green fluorescence intensity ratio (J-aggregates/monomers ratio) suggests mitochondrial depolarization. Briefly, the OC cells were seeded at a density of 6 × 10^5^ cells/well with specified media and placed for 16–18 h for cell attachment on the plate bottom in the 37 °C incubator equipped with a humidified atmosphere mixing with 5% CO_2_ and 95% air. After treatment with indicated pardaxin concentrations, the cells were added to JC-1 working solution, incubated for 20 min at 37 °C, and washed with HBSS solution. Following the removal of the media, the OC cells were detached and resuspended in 1 mL PBS. A CytoFLEX flow cytometer from Beckman Coulter (Southfield, MI, USA) was used to record red and green JC-1 fluorescence intensity. At least 2 × 10^4^ cells/group were analyzed using Beckman software CytExpert 2.0, and red/green fluorescence intensity ratio calculated accordingly.

### 2.10. Measurement of Acidic Vesicular Organelles (AVOs)

The OC cells were seeded at a density of 4 × 10^5^ cells/well with specified media and placed for 16–18 h for cell attachment on the plate bottom in the 37 °C incubator equipped with a humidified atmosphere mixing with 5% CO_2_ and 95% air. They were then challenged with pardaxin (0–5 μM) for 24 h at 37 °C. Subsequently, the cells were washed with PBS prior to adding acridine orange at 1 µg/mL and incubated for 15 min at 37 °C. Following that, the OC cells were harvested, washed with PBS, and diluted into 1 mL cold PBS. The Beckman Coulter cytometer was used for conducting the AVO experimentation. The data were analyzed using the software of Cell Lab Quanta™ SC. At least 10^4^ cells /sample were analyzed.

### 2.11. Western Blot Analysis

Before Western blotting, the OC cells were challenged with pardaxin (0, 0.1, 1, 2.5, and 5 μΜ) for 24 h. Both floating and attached OC cells were collected and washed with ice-cold PBS, followed by adding lysis buffer with RIPA reagent of Sigma-Aldrich (St. Louis, MI, USA) and incubating for 30 min at 4 °C prior to centrifugation at 13,500 rpm for 15 min. Utilizing 8–12 % SDS-PAGE, the resultant proteins were separated and then transferred to polyvinylidene fluoride (PVDF) membrane (Millipore, Burlington, MA, USA) which was soaked in 5% milk-containing Tris-buffered saline or Tween 20 to block the membrane for 1 h. Afterwards, it was incubated overnight with primary antibodies designed to capture the proteins of interest at 4 °C overnight (Supplementary Table S1). Following incubation with the secondary antibody linked with horseradish peroxidase for 60 min at 25 °C, the signals of interest from the membrane were detected using a solution of enhanced chemiluminescence (ECL-kit; Millipore). The PVDF membranes were reblotted with either β-actin or GADPH antibodies as the internal control, as indicated in the respective figures. Gel bands and images were captured using UVP BioChemi imaging (UVP LLC, Upland, CA, USA). LabWorks 4.0 software (UVP LLC) was used for relative densitometric quantification of protein bands. 

### 2.12. Measurement of Mitochondrial Respiratory Functions

A Seahorse XF24 Extracellular Flux Analyzer purchased from Seahorse Bioscience (Chicopee, MA, USA) was used to detect consumption of oxygen in a cell. The OC cells were seeded in Seahorse cell culture 24-well plates (1 × 10^5^ cells/well) with specified media and placed for 16–18 h for cell attachment on the plate bottom in the 37 °C incubator equipped with a humidified atmosphere mixing with 5% CO_2_ and 95% air. The culture media were then replaced with pardaxin-containing medium at 0, 0.1, 1, 2.5, and 5 μΜ for 24 h of incubation. Following rinsing the OC cells with 1 mL of Seahorse medium (sodium bicarbonate-free DMEM), 675 μL of the Seahorse medium were added to each well. Four measurements of the basal OCR were recorded and averaged under basal conditions, then sequentially adding 1 μM of oligomycin, 250 nM of FCCP, and 2 μM of rotenone. Subsequently, the OC cells were washed with ice-cold PBS and lysis buffer with RIPA reagent for 30 min at 4 °C, followed by centrifugation at 13,500 rpm for 15 min. The concentrations of proteins from the resultant supernatant were determined with a DC protein assay kit (Bio-Rad, CA, USA). A standard curve of protein concentration was generated using bovine serum albumin. To compare results, data were expressed as OCR in pmol/min/mg protein and ECAR in mpH/min/ mg protein, and their values were calculated after normalizing with the protein concentration.

### 2.13. Mitochondrial Morphology

To prepare transfection, a mixture of 0.5 mg of pcDNA 3.1(+)-DsRed2-Mito-7 and 1 mL of Lipofectamine 2000 (Invitrogen, Carlsbad, CA, USA) in a total volume of 100 μL was incubated for 20 min to allow the formation of DNA-Lipofectamine complexes. Each well containing 1 × 10^5^ cells was added to the solution of the complexes that were diluted in 0.5 mL of Opti-MEM medium, mixed gently, and incubated in a CO_2_ incubator at 37 °C for 4–6 h. After a few days of cell recovery, the OC cells were then cultured in Minimum Essential Medium Eagle Alpha Modification medium supplemented with 10% FBS in the CO_2_ incubator at 37 °C. After overnight incubation, the cancer cells were then challenged with pardaxin (0, 0.1, 1, 2.5, and 5 μM) in serum-free medium for additional 16 h, and added 4% paraformaldehyde for fixation. After the drug treatment, the nuclei were DAPI stained. After being processed with Sigma-Aldrich’s mounting media, the slides were viewed using a Leica Microsystems TCS SP5 II confocal microscope at a magnification of 2000× (Wetzlar, Germany). A semi-quantitative analysis scale was employed to evaluate the degree of mitochondrial network formation using the procedures as described in the literature [38]. The values for scoring mitochondrial network are a range of 0 to 3 (0 represents fragmented or punctuated network, while 3 stands for well-defined network). The score of 30–50 cells was evaluated independently by two researchers, and the readings were averaged.

### 2.14. Statistical Analysis

Statistical analysis was carried out with SPSS version 13.0, Windows version. The results of Student’s *t*-test were expressed as means ± SE from three independent trials, where * *p* < 0.05 and ** *p* < 0.01 are considered to be statistically significant.

## 3. Results

### 3.1. Pardaxin Significantly Induces Apoptosis via DNA Fragmentation and Caspase Activation

**Figure 1:**
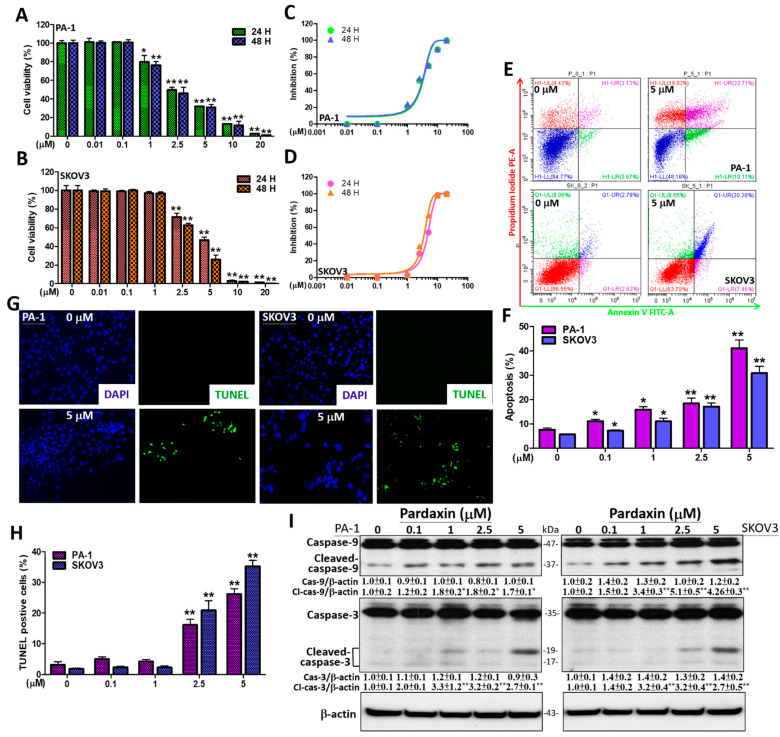
The effects of pardaxin on viability and DNA fragmentation in PA-1 and SKOV3 cells. (**A**) PA-1 cells were challenged with pardaxin at concentrations of 0–20 μM for 24 and 48 h prior to the MTT assay. The results are shown as the percentage of viable cells in comparison with pardaxin-untreated cells (0 μM); (**B**) SKOV3 cells were treated with pardaxin at 0–20 μM for 24 and 48 h and evaluated by the MTT assay. The results are demonstrated as the percentage of viable cells in comparison with pardaxin-untreated cells (0 μM); (**C**) IC_50_ values of PA-1 cells were, respectively, determined to be ~3.1 and ~3.0 μM after 24 and 48 h of pardaxin exposure; (**D**) IC_50_ values of SKOV3 cells were respectively determined to be ~4.6 and ~3.5 μM after 24 and 48 h of pardaxin incubation; (**E**) Flow cytometry with staining of Annexin V and PI was performed to evaluate pardaxin-induced apoptosis. The left charts represent the results for untreated cells, whereas the right charts show the results at 5 μM pardaxin. In the chart, the upper right quadrant (Annexin V^positive^/PI^positive^) indicates the number of apoptotic cells and the lower right quadrant indicates the number of early apoptotic cells. (**F**) Quantitation of Annexin V^positive^/PI^positive^ and Annexin V^positive^/PI^negative^ regions at concentrations of 0–5 μM of pardaxin. (**G**) The TUNEL assay shows apoptotic bodies (green immunofluorescence) after treatment with 0 and 5 μM of pardaxin for 24 h. DAPI staining (blue) was completed to observe cell DNA/nuclei using a laser confocal microscope at a magnification of 200×. (**H**) Quantification of TUNEL positive cells. (**I**) The PA-1 and SKOV3 cells were challenged with 0–5 μM of pardaxin for 24 h. Protein bands from the Western blot showing the bands for caspases-3/9, cleaved forms of caspases-3/9 as well as the internal control β-actin. Full, uncropped Western blot images and bar charts of the quantified protein values are presented in Supplementary Figure S1. Each bar represents the result of mean ± SE determined from three independent trials. Student’s *t*-test was used for analysis in which * *p* < 0.05 and ** *p* < 0.01 show statistical significance compared with the control.

To measure cell viability following pardaxin treatment, a range of concentrations (0, 0.01, 0.1, 1, 2.5, 5, 10, and 20 μM) were evaluated in PA-1 and SKOV3 cells using the MTT assay. After a 24 h incubation in PA-1 cells, the percentage of cell viability at 0, 0.01, 0.1, 1, 2.5, 5, 10, and 20 μM of pardaxin was 100 ± 6.9, 101 ± 9.0, 100.9 ± 0.7, 79.9 ± 16.5, 49.6 ± 7.6, 32.1 ± 1.31, 13.3 ± 0.6, and 2.7 ± 0.4, respectively, whereas in SKOV3 cells, the percentages were 100 ± 13.2, 99.2 ± 2.9, 99.1 ± 1.8, 97 ± 4.5, 71.6 ± 9.6, 46.9 ± 7.1, 3.1 ± 1.5, and 1.4 ± 0.5, respectively. The same pardaxin concentrations were also evaluated at 48 h in both OC cells. The half-maximal inhibitory concentration (IC_50_) values for pardaxin were 4.6–3.0 μM after 24 and 48 h, respectively (Figure 1A–D, Table 1). Hence, we subsequently applied a fixed concentration of 5 μM (~IC_50_ value) or a lower range of 0–5 μM to study the effects of pardaxin. To determine the mechanism of cell death induced by pardaxin, flow cytometric studies were implemented to analyze Annexin V and PI-stained OC cells. In the Annexin V/PI chart, the lower right quadrant (Annexin V^positive^/PI^negative^) represents a group of early apoptotic cells, whereas the upper right quadrant (Annexin V^positive^/PI^positive^) indicates a group of late apoptotic cells [36]. Figure 1E shows a typical transfer of apoptotic cells (early and late) from the left to the right quadrants in PA-1 and SKOV3 cells after 0 or 5 μM pardaxin treatment for 24 h. The results indicated that the numbers of apoptotic cells were significantly elevated to 41.1 ± 7.5% and 30.9 ± 4.8% at 5 μM pardaxin compared with the untreated controls (7.6 ± 1.4% and 5.6 ± 0.3%, respectively, Figure 1F). After treatment with 0 or 5 μM pardaxin, a TUNEL assay using immunofluorescence staining in both cell lines revealed DNA fragmentation (green fluorescence) at a magnification of 200×. DAPI was used to stain the nuclei (blue fluorescence, Figure 1G). The results indicated that TUNEL-positive cells induced DNA fragmentation after treatment with 5 μM pardaxin, which significantly increased to 26.2 ± 5.5% (PA-1) and 35.3 ± 6.0% (SKOV3) compared with the controls (3.1 ± 3.2% and 1.8 ± 0.7%, respectively, 0 μM pardaxin, Figure 1H). Western blot analysis was used to analyze the expression of cleaved caspases-3/9, which was normalized to β-actin expression in both cell lines (Figure 1I). Pardaxin treatment between 0–5 μM significantly increased the amount of cleaved caspase-9 (~1.7-fold) and cleaved caspase-3 (~2.7-fold) in PA-1 cells after challenged with 5 μM pardaxin for 24 h, whereas the relative values measured in SKOV3 cells were ~4.3-fold and ~2.7-fold, respectively (Figure 1I). However, pardaxin did not affect total caspase-3/9 protein expression in the two cell lines. Collectively, the cell viability, Annexin V/PI detection, TUNEL assay, and Western blot findings indicate that pardaxin treatment significantly enhanced caspase-associated apoptosis in PA-1 and SKOV3 cells, which suggests that pardaxin induces cell death in epithelial teratocarcinoma and adenocarcinoma subtypes of OC.

### 3.2. Intracellular and Mitochondrial ROS (mtROS) Levels Are Augmented, Whereas the Mitochondrial Membrane Potential (ΔΨ) Is Reduced with Altered Expression Profiles of Bcl-2 Family Proteins after Pardaxin Treatment

**Figure 2:**
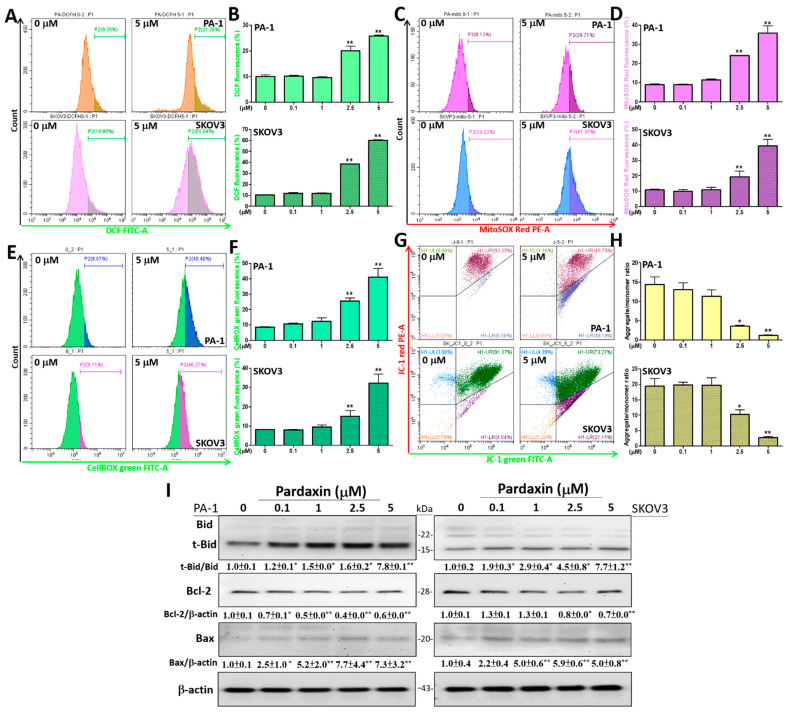
Pardaxin induces mitochondrial and intracellular ROS production, the disruption of mitochondrial membrane potential, and expression of apoptosis-related proteins in PA-1 and SKOV3 cells. (**A**) The measured fluorescence intensity of DCF for intracellular ROS at 0 or 5 µM pardaxin for 24 h; (**B**) quantitation of DCF accumulation by analyzing the selected range (5 × 10^3^–10^6^) of univariate histograms; (**C**) the measured fluorescence intensity of MitoSOX^TM^ Red for detecting mitochondrial O_2_^•−^ at 0 or 5 µM pardaxin for 24 h; (**D**) quantification of O_2_^•−^ amount in mitochondria by analyzing the selected range (5 × 10^2^–10^5^) of univariate histograms; (**E**) the measured fluorescence intensity of CellROX^®^ Green for detecting mitochondrial and nuclear O_2_^•−^ and ^•^OH at 0 or 5 µM pardaxin for 24 h; (**F**) quantification of O_2_^•−^ and ^•^OH in cancer cells by analyzing the selected range (5 × 10^4^–10^6^) of univariate histograms; (**G**) cancer cells were treated with or without 5 μM of pardaxin for 24 h. Changes in ΔΨ were measured using flow cytometry and JC-1 dye with a decrease in red fluorescence indicating mitochondrial depolarization; (**H**) quantitation of JC-1 signals by analyzing the selected range of the quadrant plot. Values from high/low ΔΨ (upper right/lower right) quadrants were used for determining the aggregated/monomer JC-1 ratios; (**I**) Western blot analyses with antibodies against apoptosis-related proteins Bid, t-Bid, Bcl-2, and Bax, and the internal control β-actin. The ImageJ software was used for densitometric analysis of intensity of each band. The densitometric values are displayed underneath the protein bands after being normalized to β-actin levels. Full, uncropped Western blot images and bar charts of the quantified protein values are shown in Supplementary Figure S2. Each bar represents the result of mean ± SE determined from three independent trials. Student’s *t*-test was performed to determine the significance where * *p* < 0.05 and ** *p* < 0.01 show statistical significance compared with the control (pardaxin-untreated cells).

Because high-energy electron flux during OXPHOS results in electron leaks, mitochondria have been considered to be the major cellular ROS producers. Therefore, we used different ROS detectors following pardaxin treatment. The fluorescent probes CM-H_2_DCFDA, MitoSOX^TM^ Red, and CellROX^®^ Green were used to detect intracellular ROS levels, mitochondrial O_2_^•−^ levels, and O_2_^•−^ and ^•^OH levels in the mitochondria and nucleus, respectively, following 24 h of pardaxin treatment at 0, 0.1, 1, 2.5, and 5 μM. The results indicated that DCF (the hydrolytic and oxidative product of CM-H_2_DCFDA) levels were significantly increased to 25.8 ± 0.5 and 60.0 ± 1.0 % at 5 μM in PA-1 and SKOV3 cells, respectively, compared with the controls (10.0 ± 1.2 and 10.2 ± 0.9%, Figure 2A,B). Similarly, mitochondrial O_2_^•−^ levels were significantly elevated based on MitoSOX^TM^ Red signals to 35.8 ± 7.4 and 39.3 ± 8.4% at 5 μM in PA-1 and SKOV3 cells, respectively, compared with the controls (8.9 ± 0.9 and 10.9 ± 0.8%, Figure 2C,D). Similarly, O_2_^•−^ and ^•^OH levels in the mitochondria and nucleus were elevated as measured by CellROX^®^ Green signals to 41.1 ± 10.9 and 32.2 ± 10.6% at 5 μM pardaxin in PA-1 and SKOV3 cells, respectively, compared with the controls (8.7 ± 0.4 and 8.2 ± 0.3 %, Figure 2E,F). Because of the high ROS levels induced by pardaxin surrounding the mitochondria, the mitochondrial membrane potential (ΔΨ) may also be affected. Accordingly, 10 μM of JC-1 dye, a specific dye for measuring relative ΔΨ, was added. In healthy cells, red fluorescent (~590 nm) J-aggregates are formed irreversibly after cationic JC-1 monomers enter the mitochondria. In contrast, in unhealthy or apoptotic cells, the original green fluorescence of the JC-1 monomers (~529 nm) remains, whereas red fluorescence decreases [39]. The results indicated that the ratios of JC-1 aggregates/monomers were decreased to 1.2 ± 0.1 and 2.8 ± 0.8% at 5 μM pardaxin in PA-1 and SKOV3 cells, respectively, compared with the controls (14.4 ± 3.8 and 19.4 ± 4.9%). This suggests a de-energized ΔΨ (Figure 2G,H).

Next, we measured the expression levels of the Bcl-2 family proteins, including Bid, the truncated form of Bid (t-Bid), Bcl-2, and Bcl-2-associated X protein (Bax). Bcl-2 is an apoptosis suppressor, whereas the other proteins are pro-apoptotic. In PA-1 cells, the t-Bid/Bid ratio and Bax levels were significantly increased to ~7.8-fold and ~7.3-fold relative to the untreated cells, respectively, following 5 μM of pardaxin treatment. In contrast, Bcl-2 levels were markedly reduced to ~0.6-fold. Similar results were observed in SKOV3 cells. The t-Bid/Bid ratio, Bax level, and Bcl-2 level were altered to ~7.7-fold, ~5.0-fold, and ~0.7-fold, respectively, relative to untreated cells (Figure 2I). Taken together, these results indicated that pardaxin enhances cellular and mtROS, and induces apoptosis through Bcl-2 family members in PA-1 and SKOV3 cells, while disrupting the mitochondrial membrane potential.

### 3.3. Autophagy-Related Acidic Vesicular Organelles Were Increasingly Detected with Upregulated Expression Levels of Autophagic Proteins after Pardaxin Treatment

**Figure 3:**
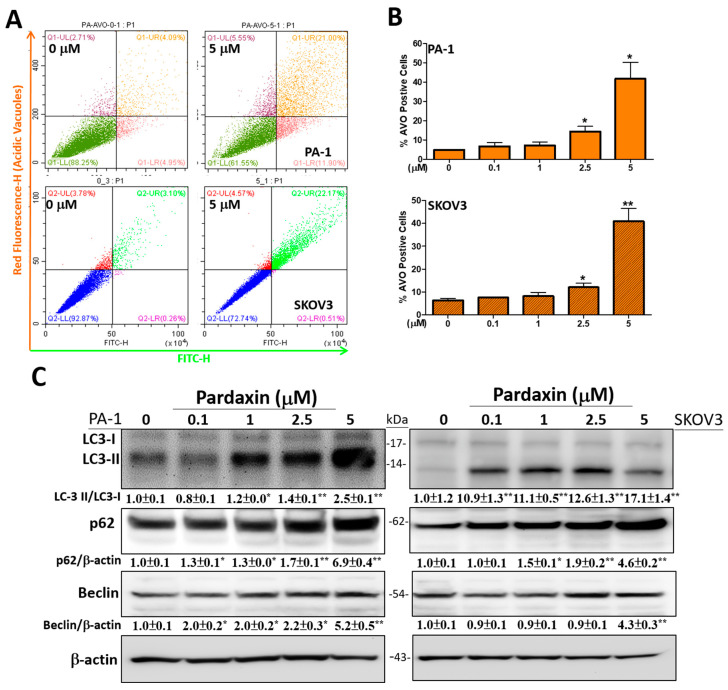
Pardaxin induces late-stage autophagy and the expression of autophagy-related proteins in PA-1 and SKOV3 cells. (**A**) The measured fluorescence intensity of AO for detecting late-stage autophagy at 0 or 5 µM pardaxin for 24 h; (**B**) Quantitation of AVO positive cells after treatment with 0–5 µM of pardaxin for 24 h were analyzed using Beckman CytoExpert flow software; (**C**) Western blot analyses with antibodies against the apoptosis-related proteins, LC3-I, LC3-II, p62, Beclin, and the internal control β-actin. The ImageJ software was used for densitometric analysis of intensity of each band. The densitometric values are displayed underneath protein bands after being normalized to β-actin levels. Full, uncropped Western blot gels and bar charts of the quantified protein values are presented in Supplementary Figure S3. Each bar represents the result of mean ± SE determined from three independent trials. Student’s *t*-test was performed to determine the significance where * *p* < 0.05 and ** *p* < 0.01 show statistical significance compared with the control (pardaxin-untreated cells).

Acridine orange (AO) is a dye that has been used for detecting autophagic events. The cytoplasm and nucleus of AO-stained cells, respectively, fluoresce as dim red bright and green colors, whereas the acidic compartments appear bright red [40,41]. The intensity of the red fluorescence is positively correlated with the degree of acidity. Autophagy is characterized by acidic vesicular organelle (AVO) formation after staining with AO. After treating with 5 µM pardaxin for 24 h, the red fluorescence signals were increased and the AVO positive cell proportion was significantly increased to 41.8 ± 19.0 and 41.0 ± 12.5% in PA-1 and SKOV3 cells, respectively, relative to the controls (4.8 ± 0.6 and 6.4 ± 1.9%, Figure 3A,B). This strongly indicates that autophagy prominently occurred in apoptotic cells. Therefore, we subsequently analyzed the expression of autophagy-associated proteins, such as Beclin, LC3-I, LC3-II, and p62 using Western blot analysis. Beclin activated autophagosome formation with proteins, such as LC3 (LC3-I, LC3-II) and p62 [20]. After treatment with 0–5 µM pardaxin for 24 h, Beclin, LC3-I/II, and p62 levels were significantly increased to 5.2 ± 0.5, 2.5 ± 0.1, and 6.9 ± 0.4 in PA-1 cells under 5 µM pardaxin, respectively, compared with the control (0 µM of pardaxin, normalized to 1.0). Similar results were also observed in SKOV3 cells in which the amounts were increased to 4.3 ± 0.3 (Beclin), 17.1 ± 1.4 (LC3-I/II), and 4.6 ± 0.3 (p62) under 5 µM pardaxin (Figure 3C).

### 3.4. The Effects of Pardaxin on Oxygen Consumption Rate, Extracellular Acidification Rate, and ETC Complex I–V Proteins of Mitochondria in PA-1 and SKOV3 Cells

**Figure 4:**
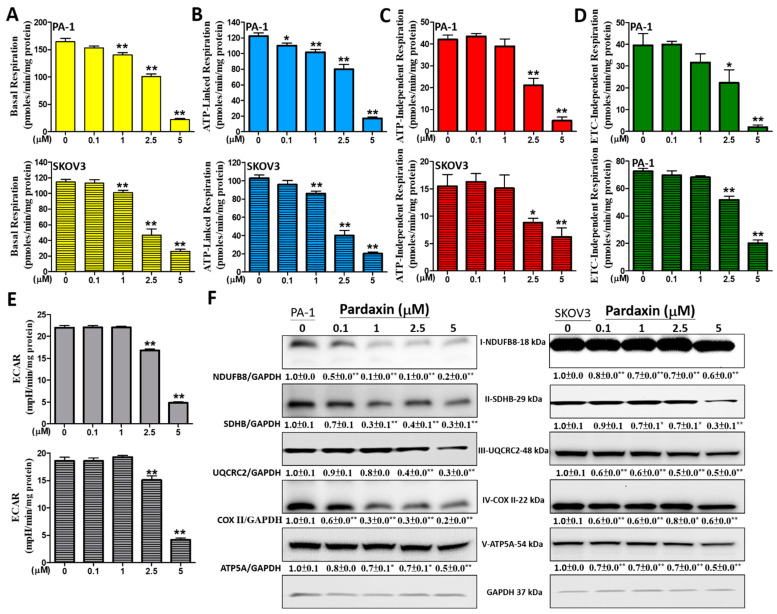
The effects of pardaxin on OCR parameters, ECARs, and five OXPHOS enzymatic complexes in PA-1 and SKOV3 cells. Both OCRs (pmoles/min/mg protein) and ECARs (mpH/min/mg protein) were measured before and after the pharmacological inhibitor solutions were added to living cells. To provide a reliable base value, four measurements were taken and averaged; subsequently, cells were sequentially and continuously injected assay reagents of the pharmacological inhibitors oligomycin, FCCP, and antimycin/rotenone. (**A**) Quantification of basal respiration OCRs; (**B**) quantification of ATP-linked respiration; (**C**) quantification of maximal respiration capacity OCRs; (**D**) quantification of proton leak respiration OCRs; (**E**) quantification of ECARs. The upper data are for PA-1 cells, whereas the lower data are for SKOV3 cells; (**F**) protein bands from the Western blot showing the effects of sinularin on the expression levels of complexes I-NDUFB8, II-SDHB, III-UQCRC2, IV-COX II, and V-ATP5A with GADPH as the blot control. Full, uncropped Western blot images and bar charts of the quantified protein values are displayed in Supplementary Figure S4. The ImageJ software was used for densitometric analysis of the protein expression levels of the abovementioned complexes and the densitometric values are displayed underneath the protein bands after being normalized with the GADPH level. Each bar represents the result of mean ± SE determined from three independent trials. Student’s *t*-test was used to determine the significance where * *p* < 0.05 and ** *p* < 0.01 show statistical significance compared with the control (pardaxin-untreated cells).

Mitochondrial respiration capacity can be measured through oxygen consumption rate (OCR) of the OXPHOS: basal, ATP-linked, ATP-independent, and ETC-independent respiration. Each OCR is derived by sequentially adding inhibitors against enzymatic complexes of the OXPHOS. Therefore, we sequentially added oligomycin for inhibiting ATP synthase (also known as complex V), FCCP for uncoupling the OXPHOS, and rotenone for inhibiting complex I. Since mitochondria are affected by pardaxin, we evaluated respiration OCR parameters following treatment with pardaxin at 0, 0.1, 1, 2.5, and 5 μM for 4 h in PA-1 and SKOV3 cells. After treatment with 5 μM pardaxin in PA-1 cells, the results indicated that mitochondrial basal respiration, ATP-linked respiration, ATP-independent respiration, and ETC-independent respiration were significantly decreased to 22.1 ± 7.2, 17.2 ± 5.2, 4.9 ± 5.8, and 2.0 ± 3.4 pmoles/min/mg protein, relative to the control at 164.2 ± 21.5, 122.1 ± 15.4, 42.0 ± 7.2, and 39.6 ± 16.9 pmoles/min/mg protein, respectively. Similarly, for SKOV3 cells, the values were significantly decreased to 26.2 ± 8.6, 20.1 ± 5.8, 6.2 ± 5.8, and 20.2 ± 8.2 pmoles/min/mg protein, relative to the control at 114.5 ± 12.4, 102.9 ± 12.0, 15.5 ± 6.4, and 72.6 ± 6.9 pmoles/min/mg protein, respectively (Figure 4A–D). The extracellular acidification rate (ECAR) measurement was subsequently performed to evaluate cellular glycolysis after 0–5 μM pardaxin treatment for 4 h in PA-1 and SKOV3 cells. Our findings demonstrated that 5 μM pardaxin administered to PA-1 and SKOV3 cells caused a significant decrease in ECAR to 4.8 ± 1.1 and 4.2 ± 1.2 mpH/min/mg of protein compared with the control (22.0 ± 1.6 and 18.6 ± 2.1 mpH/min/mg of protein) (Figure 4E). Additionally, the expression levels of the subunits of enzymatic complexes I to V were also evaluated using Western blot analyses after 0–5 μM pardaxin treatment for 4 h in PA-1 and SKOV3 cells. The subunits of complexes I, II, III, IV, and V used for Western blot analyses were NDUFB8, SDHB, UQCRC2, COX II, and ATP5A, respectively. In PA-1 cells, the results indicated that the expression of the abovementioned subunits were significantly lower after treatment with 5 μM of pardaxin, to ~0.2, ~0.3, ~0.3, ~0.2, and ~0.5-fold compared with the control, respectively (Figure 4F). In SKOV3 cells, they were significantly decreased to ~0.6, ~0.3, ~0.5, ~0.6, and ~0.5-fold compared with the control, respectively (Figure 4F). Collectively, these observations indicate that pardaxin significantly decreases mitochondrial respiration, glycolytic rate, and the function of five OXPHOS complex subunits, causing mitochondrial dysfunction in both cell lines.

### 3.5. Pardaxin Alters Mitochondrial Morpholgy by Regulating the Expression Levels of Mitochondrial Fission and Fusion Proteins

**Figure 5:**
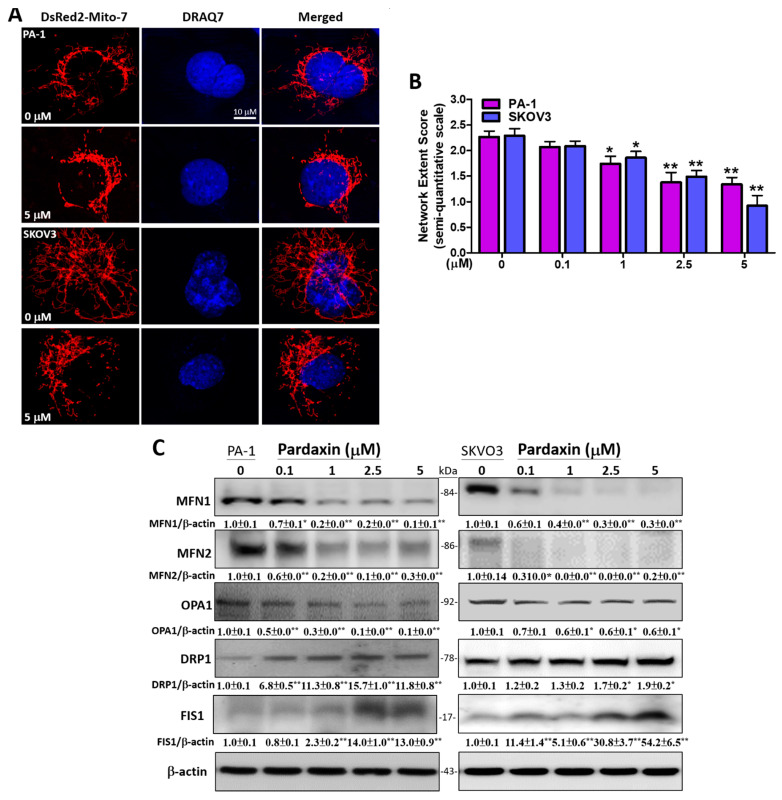
The effects of pardaxin on mitochondrial morphology and the expression of fission and fusion-related proteins in PA-1 and SKOV3 cells after being treated with 0 or 5 µM of pardaxin. (**A**) The fluorescent mitochondrial network and nuclei following the transfection of DsRed2-Mito-7 (red florescence) plasmid and DRAQ7 staining, respectively, at a magnification of 2000×; (**B**) the semi-quantitative scale values of mitochondrial network were averaged from 20–30 cells in high-power fields at a magnification of 2000×; (**C**) protein bands from the Western blot presenting the expression of MFN1/2, L-/S-OPA1, DRP1, FIS1, and the internal control β-actin. Full, uncropped Western blot images and bar charts of the quantified protein values are presented in Supplementary Figure S5. The ImageJ software was used for densitometric analysis of the protein expression levels, and the densitometric values are displayed underneath the protein bands after normalizing to corresponding β-actin levels. Each bar represents the result of mean ± SE determined from three independent trials. Student’s *t*-test was used for analysis where * *p* < 0.05 and ** *p* < 0.01 show statistical significance compared with the control.

Mitochondrial morphology, i.e., fission and fusion, is mediated by ROS and mitochondrial ATP production. During mitochondrial fusion, both mitochondrial OXPHOS and ATP production are augmented, which may induce hyperfusion of the mitochondrial network to prevent metabolic defects and death. In contrast, mitochondrial fission causes ATP consumption and OXPHOS defects, leading to mitochondrial fragmentation, which renders cells susceptible to apoptosis [42,43]. Here, we evaluated the effect of pardaxin on mitochondrial morphology in PA-1 and SKOV3 cells transfected with DsRed2-Mito-7 and stained with the fluorescent DNA, dye DRAQ7^TM^. Our findings indicate that mitochondrial network was fragmented after 5 μM pardaxin were incubated for 16 h in both cell lines (Figure 5A). A semi-quantitative analytical scale was applied to assess the degree of mitochondrial network formation. The findings showed that mitochondrial network was disrupted by increasing concentrations of pardaxin, with significant decreases to 1.3 ± 0.4 and 0.9 ± 0.6 at 5 μM in PA-1 and SKOV3 cells, respectively, compared with the controls (2.3 ± 0.4 and 2.3 ± 0.4) (Figure 5B). The expression levels of mitochondrial fusion-related proteins, MFN1/2 and OPA1, and fission-associated proteins, DRP1 and FIS1, were also evaluated by Western blot analysis after 0–5 μM pardaxin treatment. In PA-1 cells, the results indicated that the fusogenic proteins were downregulated with MFN1 showing ~0.1-fold, MFN2 ~0.3-fold, and OPA1 ~0.1-fold compared with the corresponding controls at 5 μM pardaxin. In contrast, these fission-related proteins were upregulated with DRP1 showing ~11.8-fold and FIS1 ~13.0-fold compared with the corresponding controls at 5 μM pardaxin. In SKOV3 cells, MFN1 was decreased to ~0.3-fold, MFN2 ~0.2-fold, and OPA1 ~0.6-fold compared with controls at 5 μM pardaxin. In contrast, DRP1 was increased to ~1.9-fold and FIS1 ~54.2-fold compared with controls at 5 μM pardaxin (Figure 5C). These results suggest that pardaxin effectively fragmentized the mitochondrial network by upregulating the expression of mitochondrial fission proteins, while downregulating fusion proteins in PA-1 and SKOV3 cells.

### 3.6. Pardaxin-Induced Apoptosis, ROS Generation, and Autophagy Is Reversed by Pretreatment with the Antixoidant N-acetylcysteine

**Figure 6:**
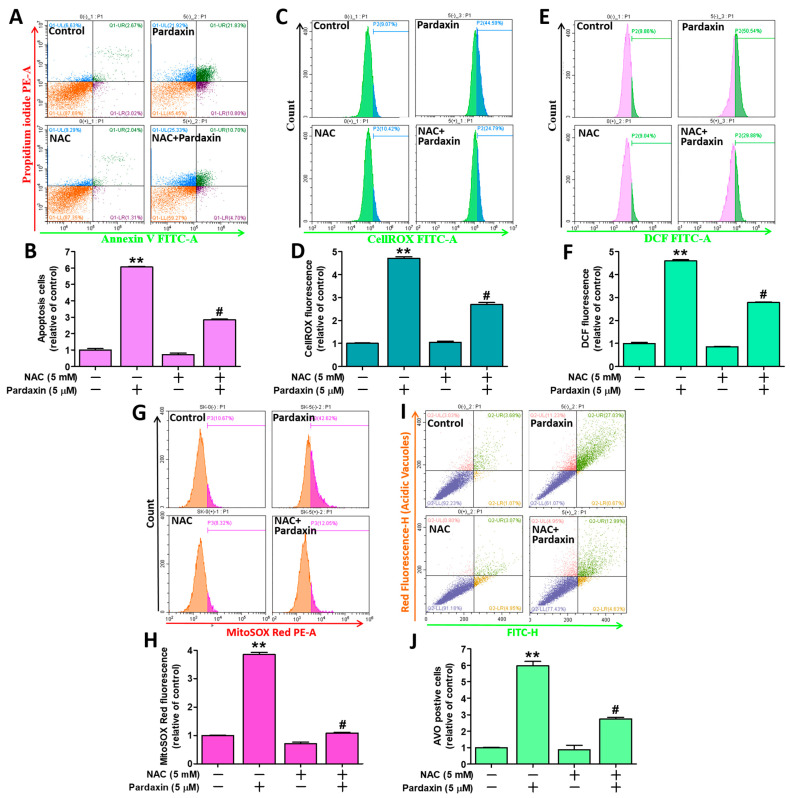
Pretreatment with antioxidant NAC rescued both intracellular and mitochondrial ROS accumulation and apoptosis induced by pardaxin in PA-1 and SKOV3 cells. (**A**) Flow cytometric analyses of cells treated with or without NAC and/or pardaxin using Annexin V and PI staining; (**B**) quantitation of Annexin V/PI detection signals; (**C**) flow cytometric analyses of cells treated with or without NAC and/or pardaxin using CellROX™ Green fluorogenic dye; (**D**) quantitation of the CellROX™ Green detection signals; (**E**) flow cytometric analyses of cells treated with or without NAC and/or pardaxin using DCF dye; (**F**) quantitation of the DCF detection signals; (**G**) flow cytometric analyses of cells treated with or without NAC and/or pardaxin using MitoSOX™ Red fluorogenic dye; (**H**) quantitation of the MitoSOX™ Red detection signals; (**I**) flow cytometric analyses of cells treated with or without NAC and/or pardaxin using AO fluorogenic dye; (**J**) quantitation of the AO detection signals. Each bar represents the result of mean ± SE determined from three independent experiments and the analyses were conducted using ANOVA method. ** *p* < 0.01 show statistical significance compared with the control group (without NAC and pardaxin), and ^#^ *p* < 0.01 relative to the experimental group with 5 μM of pardaxin alone.

The reducing agent, *N*-acetylcysteine (NAC), acts as an antioxidant within cells to deplete ROS [44]. We pretreated PA-1 and SKOV3 cells with or without 5 mM NAC for 2 h prior to challenging with or without 5 μM pardaxin. Following 24 h incubation in the incubator, the OC cells were stained with Annexin V/PI, CellROS^TM^ Green, CM-H_2_DCFDA, MitoSOX^TM^ Red, or AO and subjected to flow cytometry for analysis. The results indicated that NAC did not cause apoptosis, and apoptotic cells were elevated significantly following pardaxin treatment, which was significantly rescued by NAC pretreatment (Figure 6A,B). Similarly, NAC significantly reduced intracellular ROS levels induced by pardaxin as evidenced by decreases in CellROS^TM^ Green, DCF, and MitoSOX^TM^ Red signals (Figure 6C,H). Similarly, autophagy-indicative bodies induced by pardaxin were significantly ameliorated following NAC treatment (Figure 6I,J). Taken together, NAC significantly reverses apoptosis, ROS levels, and autophagy induced by pardaxin, which confirm ROS as the principle underlying factor that causes the above effects.

## 4. Discussion and Conclusions

Most OC types are epithelial and approximately two-thirds of ovarian epithelial cancer patients are diagnosed at stage III or IV [45]. In the present study, we used epithelial OC cell lines, PA-1 and SKOV3, which are considered a teratocarcinoma and adenocarcinoma, respectively. Since these two cell lines are undifferentiated [46,47], they are considered to be highly malignant. In addition, the epithelial characteristics render them with the ability to undergo epithelial-to-mesenchymal transition to acquire cancer stemness characteristics by upregulating neural cadherin (N-cadherin), vimentin, and fibronectin, while downregulating epithelial cadherin (E-cadherin) [48,49], as evidenced by Western blot analyses [50]. These malignant characteristics may contribute to resistance to platinum-based therapy for both OC cell lines [51].

Traditionally, most new cancer drugs are primarily sourced from the development of natural products, with more than 60% of the FDA-approved drugs from terrestrial animals and plants, but relatively few from marine creatures [52,53]. In particular, approximately 75% of the anticancer chemotherapeutic agents are derived from natural products or their derivatives [54]. Marine creatures represent a treasure trove of natural products since ocean environments are usually extreme with high salinity, high pressure, and hypoxia, resulting in a diverse structure of secondary metabolites that differ from terrestrial species. However, marine-derived resources have not been studied exhaustively. The National Cancer Institute has even suggested that the ocean is an important source of lead compounds [55]. Therefore, we have evaluated pardaxin in the context of anti-OC drug development using PA-1 and SKOV3 cells.

**Figure 7:**
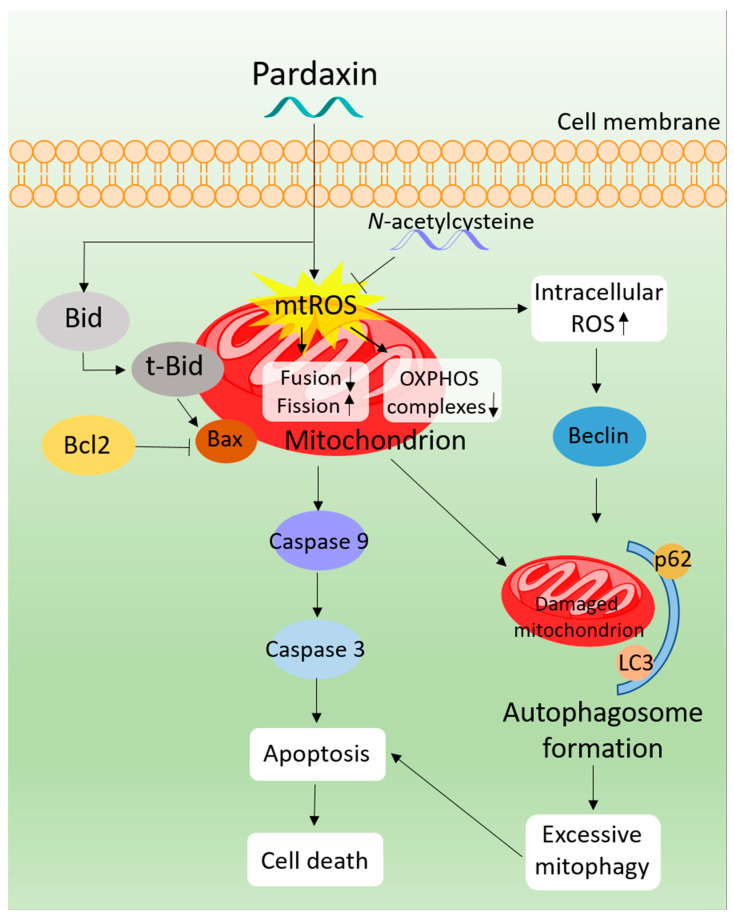
Proposed mechanisms of pardaxin in PA-1 and SKOV3 cells.

The 33-amino acid pardaxin compound exhibited cytotoxic activity against PA-1 and SKOV3 cells. Since pardaxin contains cationic and amphipathic amino acids, it can readily interact with the anionic membranes of tumor cells, which enhances entry into the cell. Pardaxin demonstrated a cytotoxic mechanism of action by inducing ROS overproduction in mitochondria, resulting in mitochondrial membrane depolarization that further caused an imbalance in mitochondrial membrane potential and initiated the activation of pro-caspases 9 and 3. Mitochondria-mediated apoptosis induced by pardaxin was also buttressed by the upregulation of t-Bid and Bax, because t-Bid is situated on the mitochondrial membrane. Since the OXPHOS enzymatic complexes were simultaneously attenuated by pardaxin-induced ROS, additional ROS generation is likely to occur that further contributes to total ROS levels. Additional studies of mitochondrial morphology showed that pardaxin fragmented the mitochondrial network, which is consistent with other ROS-generating marine peptides, such as TP3 [36], although some types of anticancer inhibitors enhance mitochondrial fusion [56]. The autophagic event was significantly promoted by pardaxin, which upregulated autophagosome-related proteins, p62, LC3, and Beclin. More specifically, mitophagic events were triggered since both mitochondrial fragmentation and autophagosome-related proteins were activated. As mentioned previously, excessive mitophagy can induce apoptosis, and as evidenced by the significant increase in fission-related and autophagosome-related proteins, pardaxin causes excessive mitophagy. The above pardaxin-induced apoptosis was rescued by treatment with the antioxidant, NAC, indicating that ROS is vital for apoptosis induction. The anticancer mechanisms of pardaxin are depicted in Figure 7.

Autophagy and apoptosis mostly occurs in a sequence in which autophagy precedes apoptosis [57] when a stimulus is not sufficiently lethal or stressful. In contrast, during excessive stimulus in duration and/or intensity (i.e., stress), apoptotic and non-apoptotic lethal programs are initiated. Usually, autophagy acts as a mechanism to cope with stimuli [58]. Nevertheless, if a cell triggers apoptosis, autophagy can be inactivated, partially because of caspase-mediated cleavage of necessary autophagy-related proteins. Beyond general circumstances, autophagy or essential proteins involved in the autophagic process may facilitate cellular demise in some specific scenarios [59], which renders the relationship between autophagy and apoptosis complicated. In the present study, we found that the AVO signal was elevated significantly after pardaxin treatment, indicating autophagy was activated. We also found that the mitochondrial network was significantly disrupted. Both findings indicate that the mitochondria were severely damaged, triggering a robust degradation process to remove the harmful organelles. These findings suggest that pardaxin-induced excessive autophagy, more specifically mitophagy, does not inhibit apoptosis as may be generally observed in cells [59]. Instead, it further reinforces apoptotic cell death, which is consistent with the findings of Chen et al. [21].

Under normal conditions, the ETC process in the OXPHOS is not considered to be ideal. Leakage of electrons from complexes I and III results in reduction of oxygen to form O_2_^•−^ in part, with an estimate that 0.2–2.0% of oxygen consumed by mitochondria is converted to O_2_^•−^ [60]. Subsequently, O_2_^•−^ is quickly converted to H_2_O_2_ by dismutases including superoxide dismutases 1 and 2. Therefore, both O_2_^•−^ and H_2_O_2_ yielded from this process are considered mtROS and the levels of H_2_O_2_ are positively correlated with O_2_^•−^. Because of its negatively charged and short half-life characteristics, O_2_^•−^ hardly passes from the mitochondrial outer membrane to the cytosol, and is thus unable to stimulate signal transduction molecules in the cytosol. Instead, O_2_^•−^ prefers having a radical–radical reaction with nitric oxide to yield an adduct of peroxynitrite within mitochondria, a harmful oxidant known to cause DNA damage, disruption of mitochondrial integrity, and detrimental modification of proteins [60]. In contrast, H_2_O_2_ is neutrally charged and more stable than O_2_^•−^, enhancing its ability to cross the mitochondrial membrane to influence intracellular components and signal transduction pathways. In the present study, we found that the O_2_^•−^ levels were elevated in mitochondria and intracellular H_2_O_2_ was positively correlated with mitochondrial O_2_^•−^ levels, which indicates that pardaxin-induced ROS are initially generated in the mitochondria.

DNA damage induced by ionizing radiation is the primary mechanism through which radiation therapy kills cancer cells. Thus, inducing additional ROS within cancer cells is beneficial for enhancing the radiotherapeutic effect. Pardaxin-induced ROS has been found in mitochondria and the nucleus, as evidenced by MitoSOX^TM^ Red and CellROX^TM^ Green fluorescence, and the resulting ROS may break DNA in an efficient manner. Besides, most tumor types reside in a complex microenvironment that is hypoxic, which impedes ROS generation by ionizing radiation, hence decreasing radiotherapeutics efficacy. A probable tactic to mitigate hypoxia is to downregulate OXPHOS capacity. This strategy is based on the idea that inhibiting OXPHOS decreases oxygen consumption by mitochondria, thus increasing oxygen availability for use in the cytosolic compartments and nearby tissues [61]. Our findings clearly indicate that pardaxin attenuates the tested mitochondrial OCR parameters in PA-1 and SKOV3 cells, which preserves oxygen in the cytosolic compartments and tumor microenvironment. Collectively, ROS overproduced by pardaxin and pardaxin-induced mitochondrial respiratory dysfunction support the use of this marine peptide as an adjuvant to radiotherapy.

## Figures and Tables

**Table 1:** IC_50_ values at 24 and 48 h incubation of pardaxin determined by MTT assay.

IC_50_ Values (μM)		
Time (h)	PA-1	SKOV3
24	3.12 ± 0.21	4.60 ± 0.17
48	3.00 ± 0.22	3.51 ± 0.14

## Data Availability

Data is contained within the article and supplementary materials.

## Supplementary Materials

The following are available online at https://www.mdpi.com/article/10.3390/antiox10121883/s1, Figure S1: Original, uncropped images of the Western blots for Figure 1I displayed in the text and results; Figure S2: Original, uncropped images of the Western blots for Figure 2I displayed in the text and results; Figure S3: Original, uncropped images of the Western blots for Figure 3C displayed in the text and results; Figure S4: Original, uncropped images of the Western blots for Figure 4F displayed in the text and results; Figure S5: Original, uncropped images of the Western blots for Figure 5C displayed in the text and results; Table S1: Information on primary antibodies used in the Western blot analysis of this study.
