# Supplementary material for: Pardaxin Activates Excessive Mitophagy and Mitochondria-Mediated Apoptosis in Human Ovarian Cancer by Inducing Reactive Oxygen Species
Source: Antioxidants (Basel). 2021 Nov 25;10(12):1883. doi: 10.3390/antiox10121883 (PMC8698909; doi:10.3390/antiox10121883)
Supplement: Supplementary file 1 [file antioxidants-10-01883-s001.zip › antioxidants-1457419-supplementary.pdf]

Pardaxin activates excessive mitophagy and mitochondria-mediated apoptosis in human ovarian cancer by inducing reactive oxygen species

Yen-Po Chen[1,2*], Po-Chang Shih[1,3*], Chien-Wei Feng[4], Chang-Cheng Wu[1,5], Kuan-Hao Tsui[6,7], You-Hsien Lin[8,9], Hsiao-Mei Kuo[1,10†] and Zhi-Hong Wen[1,11†]

[1]Department of Marine Biotechnology and Resources, National Sun Yat-Sen University, Kaohsiung 80424, Taiwan; E-Mails: D075020002@nsysu.edu.tw (Y.-P.C.); po-chang.shih.14@ucl.ac.uk (P.-C.S.); nhk@ngh.com.tw (C.-C.W.); wzh@mail.nsysu.edu.tw (Z.-H.W.); Hsiaomeikuo@g-mail.nsysu.edu.tw (H.-M.K.)

[2]Department of Obstetrics and Gynecology, Kaohsiung Armed Forces General Hospital, Kaohsiung 80284, Taiwan; E-Mails: D075020002@nsysu.edu.tw (Y.-P.C.)

[3]Department of Neurosurgery, Kaohsiung Chang Gung Memorial Hospital, Kaohsiung 83301, Taiwan; E-Mails: po-chang.shih.14@ucl.ac.uk (P.-C.S.)

[4] Department of Obstetrics and Gynecology, Kaohsiung Medical University Hospital, Kaohsiung Medical University, Kaohsiung 80756, Taiwan; E-Mails: 1080532@kmuh.org.tw (C.-W.F.)

[5]Department of Obstetrics and Gynecology, Zouying Branch of Kaohsiung Armed Forces General Hospital, Kaohsiung 81342, Taiwan; E-Mails: nhk@ngh.com.tw (C.-C.W.)

[6]Department of Obstetrics and Gynecology, Kaohsiung Veterans General Hospital, Kaohsiung 81341, Taiwan; E-Mails: khtsui@vghks.gov.tw (K.-H.T.)

[7]Department of Obstetrics and Gynecology and Institute of Clinical Medicine, National Yang-Ming University, Taipei 11221, Taiwan; E-Mails: khtsui@vghks.gov.tw (K.-H.T.)

[8]Department of Internal Medicine, Kaohsiung Municipal Ta-Tung Hospital, Kaohsiung 80145, Taiwan; E-Mails: hugoyl@kmu.edu.tw (Y-H.L.)

[9]Department of Medicine, College of Medicine, Kaohsiung Medical University, Kaohsiung 80708, Taiwan; E-Mails: hugoyl@kmu.edu.tw (Y-H.L.)

[10]Center for Neuroscience, National Sun Yat-sen University, Kaohsiung 80424, Taiwan; E-Mails: Hsiaomeikuo@g-mail. nsysu.edu.tw (H.-M.K.)

[11]Institute of Medical Science and Technology, National Sun Yat-Sen University, Kaohsiung, Taiwan; E-Mails: wzh@mail.nsysu.edu.tw (Z.-H.W.)

* These authors contributed equally to this work.

†Correspondence:Hsiaomeikuo@g-mail.nsysu.edu.tw (H.-M.K.); wzh@mail.nsysu.edu.tw (Z.-H.W.)
TEL: +886-7-5252000 #5038
FAX: +886-7-5252021

1. Supplementary Table

Supplementary Table S1. Information on primary antibodies used in the western blot analysis of this study.

Antigen	Host	Catalogue	Clonality	Supplier	Dilution ratio
Anti-Caspase 3	Rabbit	14420	Polyclonal	Cell Signaling	1:1000
Anti-Caspase 9	Rabbit	9502	Polyclonal	Cell Signaling	1:1000
Anti-Bax	Rabbit	2772S	Polyclonal	Cell Signaling	1:1000
Anti-Bcl-2	Mouse	15071	Monoclonal	Cell Signaling	1:1000
Anti-Bid	Rabbit	2002S	Polyclonal	Cell Signaling	1:1000
Anti-Beclin	Rabbit	11306-1-ap	Polyclonal	Proteintech	1:1000
Anti-LC3I/II	Rabbit	Ab58610	Polyclonal	Abcam	1:1000
Anti-p62/SQSTM1	Rabbit	18420-1-ap	Polyclonal	Cell Signaling	1:1000
Total OXPHOS Human WB Antibody Cocktail	Mouse	ab110411	Monoclonal	Abcam	1:1000
Anti-SDHB	Mouse	ab14714	Monoclonal	Abcam	1:1000
Anti-UQCRC2	Mouse	ab14745	Monoclonal	Abcam	1:1000
Anti-ATP5A	Mouse	ab14748	Monoclonal	Abcam	1:1000
Anti-MFN1	Rabbit	ABC41	Polyclonal	Millipore	1:1000
Anti-MFN2	Rabbit	ABC42	Polyclonal	Millipore	1:1000
Anti-OPA1	Rabbit	ABN95	Polyclonal	Millipore	1:1000
Anti-FIS1	Rabbit	SC-98900	Polyclonal	Santa Cruz	1:500
Anti-DRP1	Rabbit	SC-32898	Polyclonal	Santa Cruz	1:500
Anti-β-actin	Mouse	A5441	Monoclonal	Sigma-Aldrich	1:5000
Anti-GAPDH	Rabbit	GTX100118	Polyclonal	GeneTex	1:5000

Supplementary Figures

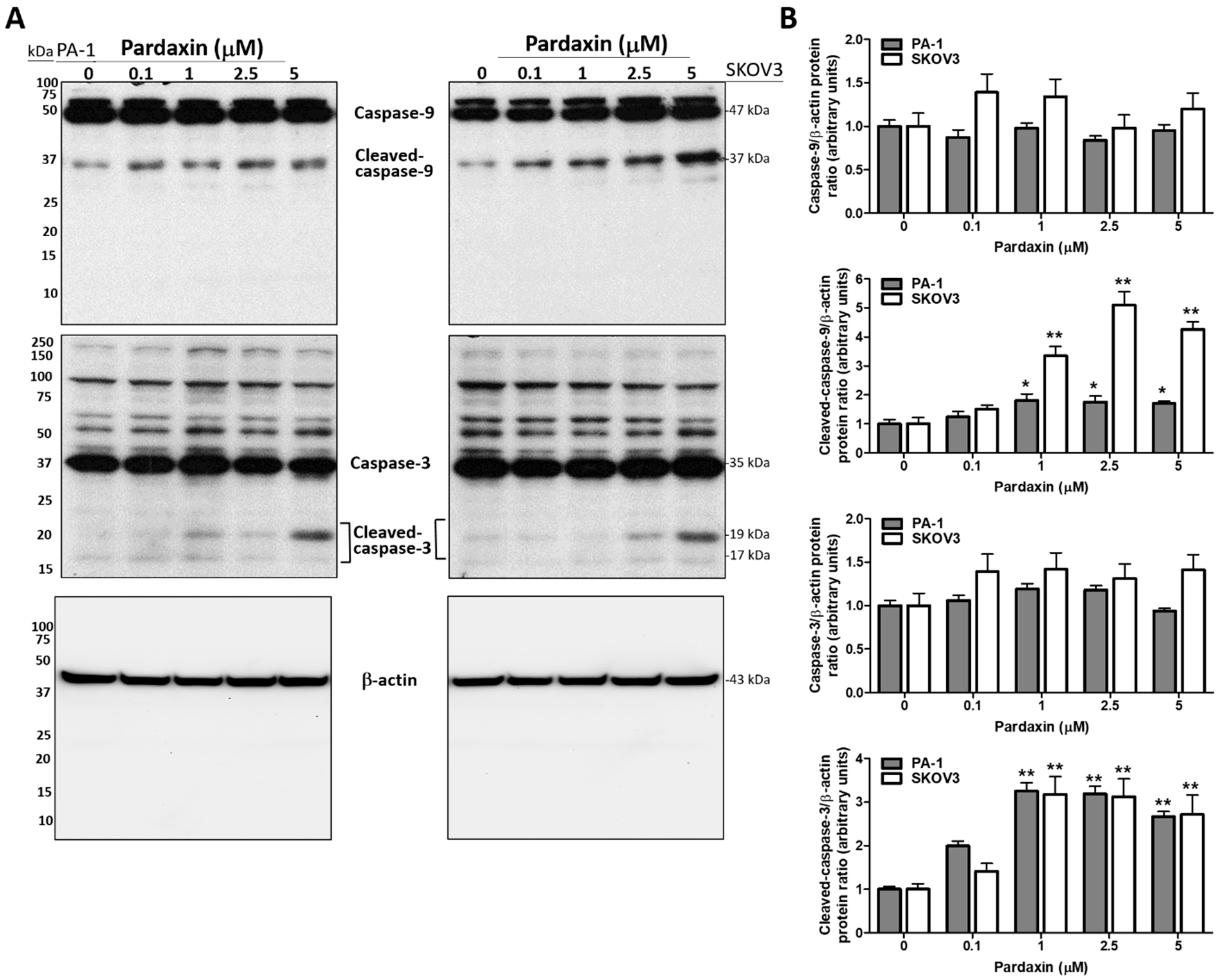

Supplementary Figure S1. Original, uncropped images of the western blots for Fig. 1I displayed in the text and results. (A) The bands of cleaved caspase-9, pro-caspase-9, cleaved caspase-3, pro-caspase-3 and β-actin, and their expected molecular weight, with β-actin used as the protein loading control in PA-1 (left) and SKOV3 cells (right). (B) Quantification of the bands of cleaved caspase-9, pro-caspase-9, cleaved caspase-3, and pro-caspase-3, after normalized with the internal control β-actin.

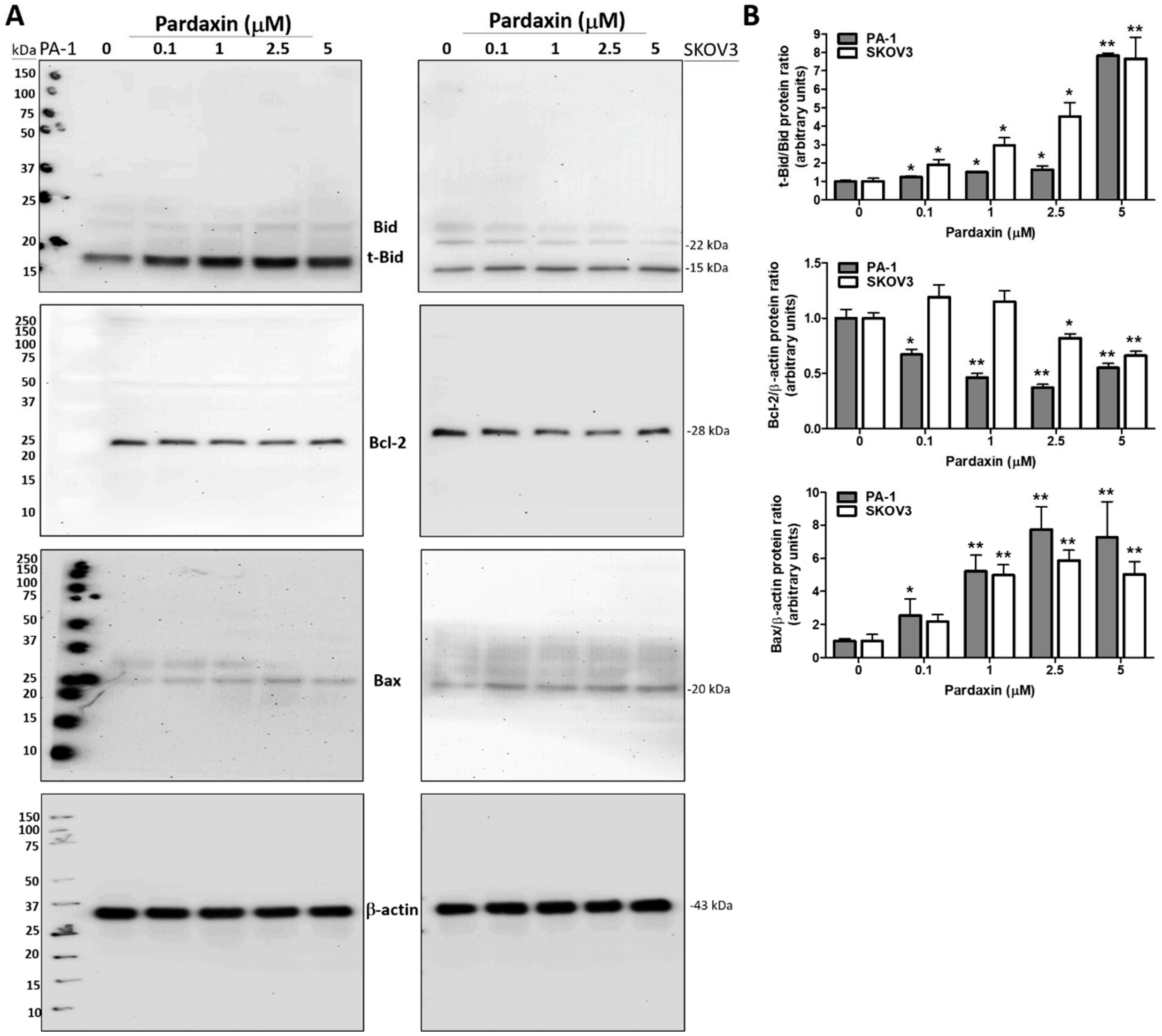

Supplementary Figure S2. Original, uncropped images of the western blots for Fig. 2I displayed in the text and results. (A) The bands of Bid, t-Bid, Bcl-2, Bax and β-actin and their expected molecular weight, with β-actin used as the protein loading control in PA-1 (left) and SKOV3 cells (right). (B) Quantification of the bands of Bid, t-Bid, Bcl-2, and Bax, after normalized with the internal control β-actin.

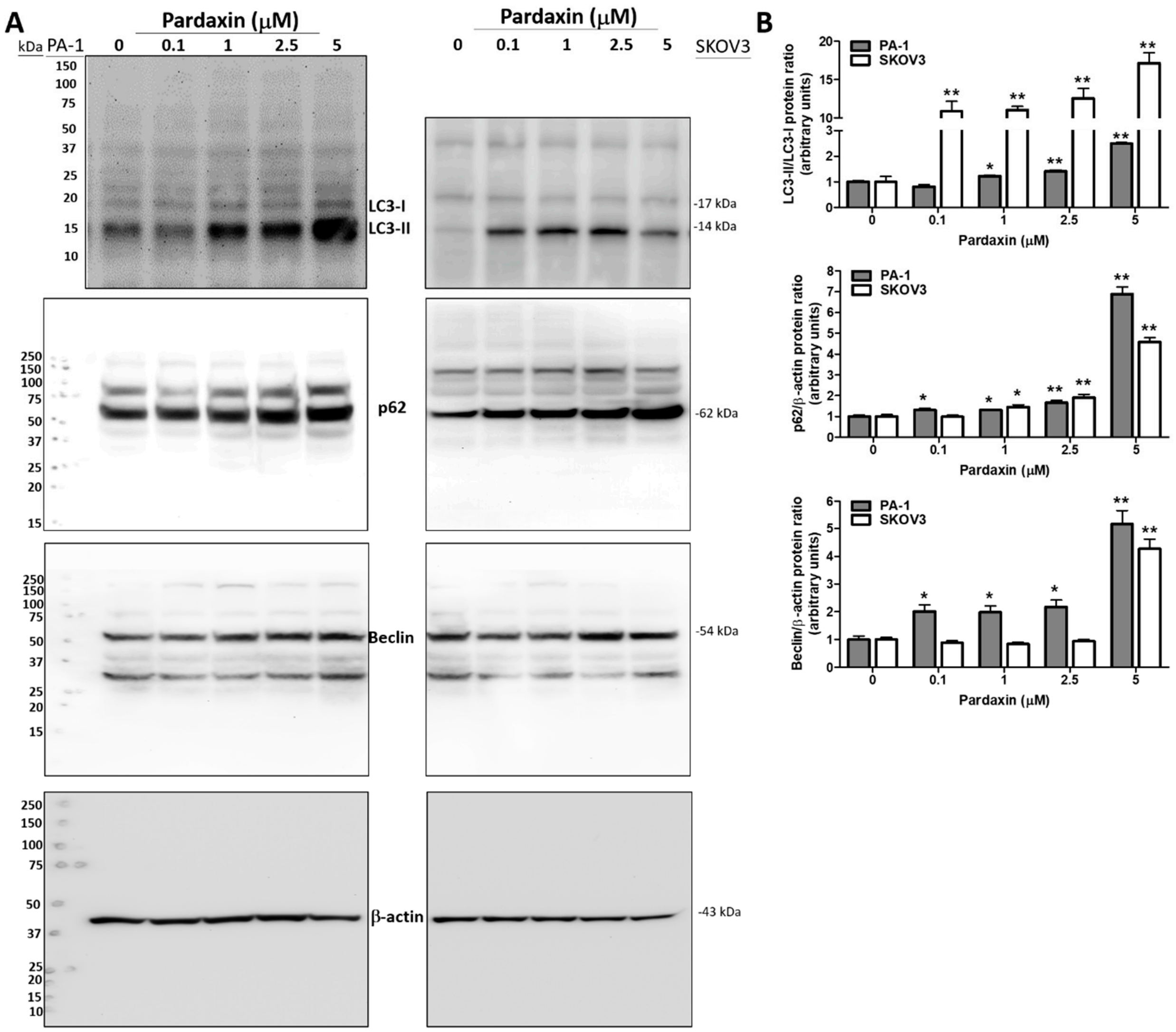

Supplementary Figure S3. Original, uncropped images of the western blots for Fig. 3C displayed in the text and results. (A) The bands of LC3-I, LC3-II, p62, Beclin and β-actin bands and their expected molecular weight, with β-actin used as the protein loading control in PA-1 (left) and SKOV3 cells (right). (B) Quantification of the bands of LC3-I, LC3-II, p62, and Beclin, after normalized with the internal control β-actin.

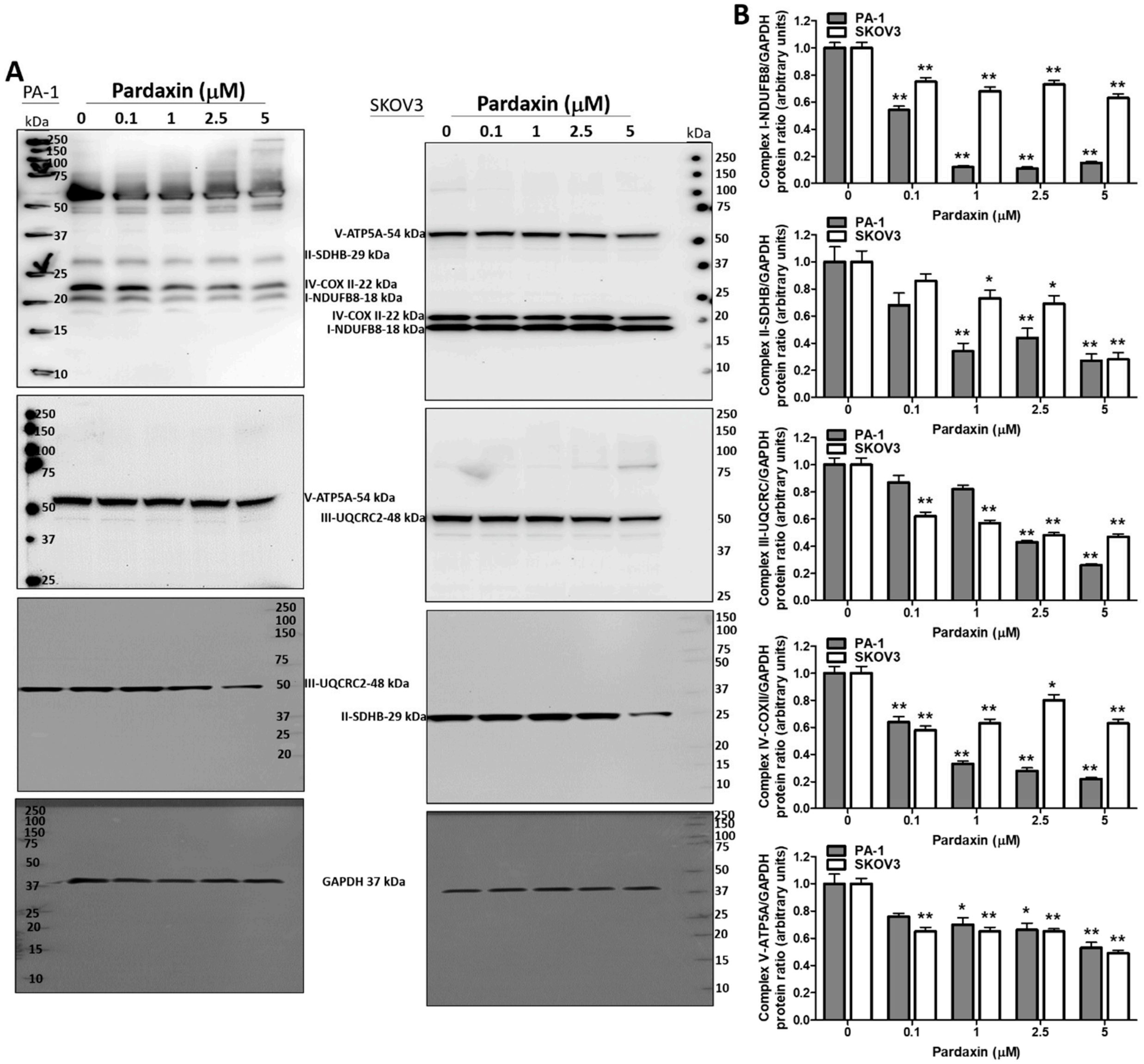

Supplementary Figure S4. Original, uncropped images of the western blots for Fig. 4F displayed in the text and results. (A) The bands of indicated OXPHOS enzymatic complexes examined using Total OXPHOS Human WB Antibody Cocktail (Cat#ab110411, Abcam, Cambridge, UK), with GADPH used as the protein loading control in PA-1 (left) and SKOV3 cells (right). (B) Quantification of the bands of the indicated OXPHOS enzymatic complexes, after normalized with the internal control GADPH.

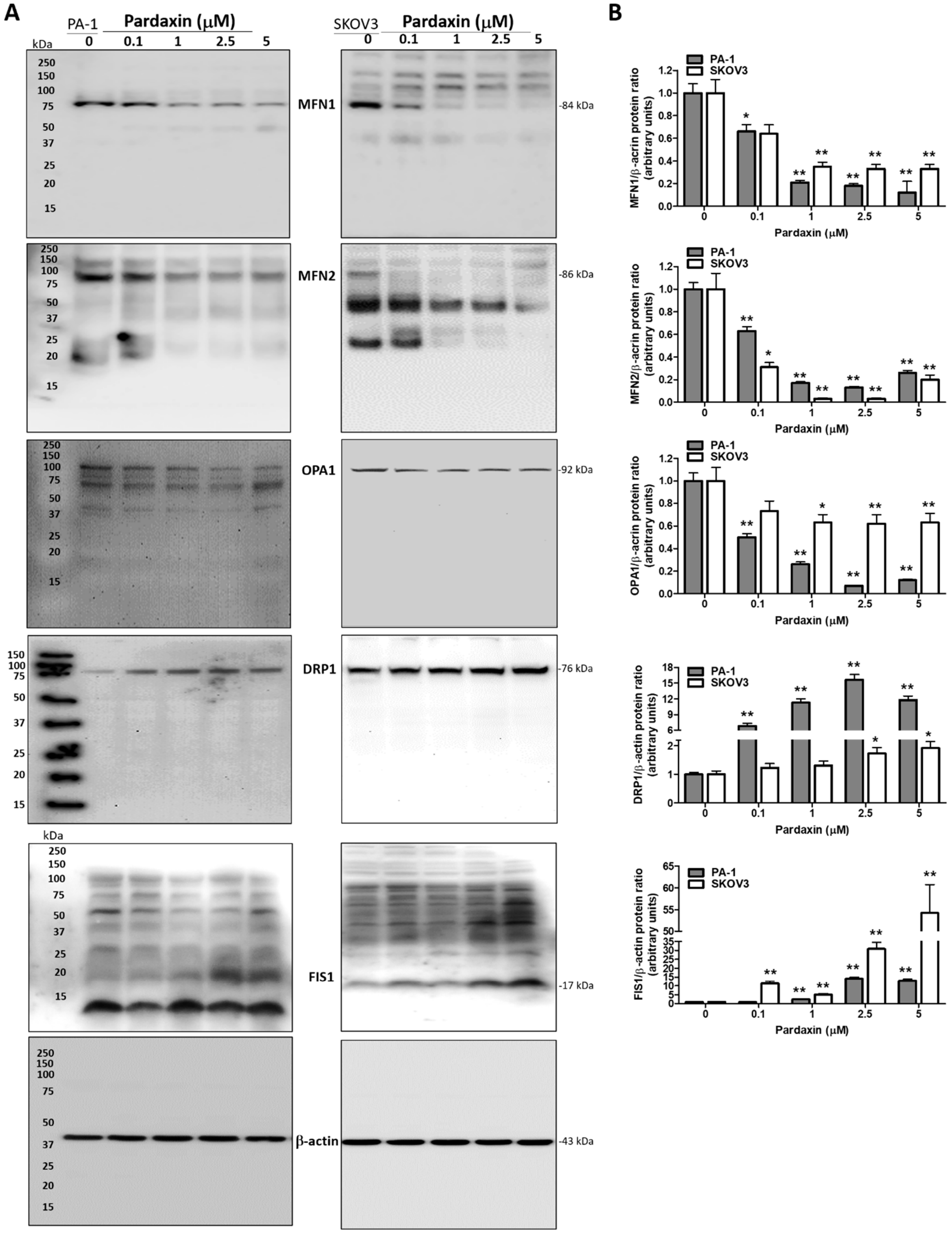

Supplementary Figure S5. Original, uncropped images of the western blots for Fig. 5C displayed in the text and results. (A) The bands of MFN1, MFN2, OPA1, DRP1, FIS1 and β-actin bands and their expected molecular weight, with β-actin used as the protein loading

control in PA-1 (left) and SKOV3 cells (right). (B) Quantification of the bands of MFN1, MFN2, OPA1, DRP1, and FIS1, after normalized with the internal control β-actin.
